# Hydrogel systems for spatiotemporal controlled delivery of immunomodulators: engineering the tumor immune microenvironment for enhanced cancer immunotherapy

**DOI:** 10.3389/fcell.2024.1514595

**Published:** 2024-12-13

**Authors:** Yanting Liu, Fang Liu, Yan Zeng, Liangbin Lin, Hui Yu, Sunfu Zhang, Wenyong Yang

**Affiliations:** ^1^ Department of Oncology, The First Affiliated Hospital of Xinxiang Medical University, Weihui, Henan, China; ^2^ Department of Neurosurgery, Department of Urology, Medical Research Center, The Second Chengdu Hospital Affiliated to Chongqing Medical University, The Affiliated Hospital of Southwest Jiaotong University, The Third People’s Hospital of Chengdu, Chengdu, China; ^3^ College of Public Health (Shenzhen), Sun Yat-sen University, Shenzhen, China; ^4^ West China School of Basic Medical Sciences and Forensic Medicine, State Key Laboratory of Biotherapy and Cancer Center, West China Hospital, Collaborative Innovation Center for Biotherapy, Sichuan University, Chengdu, China; ^5^ Obesity and Metabolism Medicine-Engineering Integration Laboratory, Department of General Surgery, The Third People's Hospital of Chengdu, The Affiliated Hospital of Southwest Jiaotong University, Chengdu, China; ^6^ The Center of Gastrointestinal and Minimally Invasive Surgery, Department of General Surgery, The Third People's Hospital of Chengdu, The Affiliated Hospital of Southwest Jiaotong University, Chengdu, China

**Keywords:** hydrogel, immunomodulator, tumor immune microenvironment, innate immunity, adaptive immunity

## Abstract

Tumor immunotherapy, modulating innate and adaptive immunity, has become an important therapeutic strategy. However, the tumor immune microenvironment’s (TIME) complexity and heterogeneity challenge tumor immunotherapy. Hydrogel is a hydrophilic three-dimensional (3D) mesh structure with good biocompatibility and drug release control, which is widely used in drug delivery, agriculture, industry, etc. Hydrogels loaded with immune cells, cytokines, immune checkpoint inhibitors, and anti-tumor drugs can achieve targeted delivery and ultimately activate the immune response in the TIME. In this review, we will summarize the components of the TIME and their immune effects, the emerging immunomodulatory agents, the characteristics and functions of hydrogels, and how hydrogels regulate innate and adaptive immune cells in the TIME.

## 1 Introduction

In recent years, the incidence and mortality of cancer have been rising all over the world. According to global statistics, there were 19.3 million new cases of cancer and nearly 10 million deaths due to cancer in 2020 (Sung et al., 2021). Tumor treatments mainly include surgery, chemotherapy, radiotherapy, immunotherapy, etc. Immunotherapy has presented an impressive anti-tumor effect in tumor therapy due to rewiring the tumor immune microenvironment (TIME) to attack cancer cells specifically (Tan et al., 2020). At present, a variety of cancer immunotherapies have been designed, including immune checkpoint blocking (ICB) therapies, cancer vaccines, agonistic antibodies, and chimeric antigen receptor T-Cell (CAR-T) immunotherapy (Tan et al., 2020; Wang and Mooney, 2018).

However, the TIME brings obstacles to the application of immunotherapy. In the TIME, there exist numerous immunosuppressive cell populations that promote tumor immune escape by directly inhibiting the function of immune cells or altering the tumor microenvironment. These immunosuppressive cells include Tregs, MDSCs, and tumor-associated macrophages (TAMs), among others. They collectively create an immunosuppressive environment by secreting immunosuppressive factors, depleting nutrients, or altering the pH of the tumor microenvironment. The extracellular matrix (ECM) of tumor tissue often exhibits excessive deposition and a “stiff” state of collagen, fibronectin, and other components, which not only restricts the infiltration of immune cells but also further inhibits their function through mechanisms such as inducing hypoxia. Additionally, abnormalities in tumor blood vessel structure constitute an important part of the physical barrier, impeding the effective delivery of immune cells and the supply of nutrients. Therefore, TMIE severely affects the efficacy of tumor immunotherapy (Tan et al., 2020; Xiao et al., 2023; Seo et al., 2022). How to precisely regulate the immune microenvironment, relieve immunosuppression, and enhance the efficacy of immunotherapy is crucial for the treatment of tumors.

The emergence of biomaterials has enabled researchers to achieve precise delivery of cytokines and targeted regulation of signaling pathways, making it possible to efficiently and low-toxically activate anti-tumor immunity (Wang and Mooney, 2018; Ruan et al., 2022; Hu et al., 2021). Hydrogel is a hydrophilic three-dimensional (3D) mesh structure of biological materials, and the water content can be as high as 90% (Xie et al., 2021; Ahmed, 2015; Cui et al., 2021). Due to the high degree of customizability and tunability, hydrogel can be designed to suit a variety of requirements. Therefore, hydrogel has a wide range of applications in many fields, including medicine (Ho et al., 2022; Khan et al., 2022). Due to biocompatibility, highly hydrophilic and porous structure, high drug-loading, low toxicity, stimuli-responsiveness, controlled release, hydrogels hold promise in overcoming immune barriers within the tumor immune microenvironment, enabling precise delivery of immunomodulatory drugs such as cytokines, as well as precise targeting of immunosuppressive cells. They have significant application prospects in the regulation of TMIE and immune activation.

Hydrogel can play a regulatory role in cell growth and differentiation, such as remodel the extracellular matrix (ECM), creating an environment conducive to cell growth and differentiation (Xiao et al., 2023; Yue et al., 2015). Hydrogel’s drug release rate can be controlled by adjusting its crosslinking degree, polymer chain length, and introducing functional groups (Teixeira et al., 2021; Ashfaq et al., 2020), which ensures its function of release immunomodulators in a controlled manner while protecting their bioactivity is highlighted (Cao et al., 2021; Almawash et al., 2022; Chin et al., 2020). What’s more, hydrogel can deliver immunomodulators or drugs for regulating tumor immunity. Hydrogel loaded with IL-4 and BMP-2 promotes macrophage differentiation into the M2 type, which can effectively regulate tumor immunity (Zou et al., 2021). GM-CSF released by an injectable heat-sensitive hydrogel mediates the recruitment, proliferation, and maturation of dendritic cells, promoting an effective immune response (Warren and Weiner, 2000; Liu et al., 2014). Another example is provided where the delivery of doxorubicin and cytosine-phospho-guanine self-crosslinking nanoparticles based on the hydrogel increases the number of CD8^+^ T cells and reduces the number of immunosuppressive cells in the TIME (Dong et al., 2020).

In this review, we initially examine the components of the tumor immune microenvironment (TIME) and their immune implications. Next, we summarize emerging immunomodulatory agents and provide an overview of hydrogels, including their classification, characteristics, and applications. Finally, we outline the primary mechanisms through which hydrogels regulate innate and adaptive immune cells, illustrating these with relevant examples to facilitate understanding of their functions.

## 2 Composition of the TIME

TIME consists of many components, including DCs, macrophages, natural killer (NK) cells, B cells, T cells, and ECM (Arneth, 2019). It is an immunosuppressive environment that benefits tumor progression. However, many components in the TIME play an active anti-tumor immune function. The components of the TIME and their immune effects are listed in Table 1 (Arneth, 2019; Bilotta et al., 2022).

**TABLE 1:** Components of the tumor immune microenvironment (TIME) and their immune effects.

Components of the TIME	Category	Immune effects
Dendritic cells (DCs)		Immune surveillance, cytokine secretion, presentation of antigens, and initiation of tumor-killing function of T cells
Macrophages	M1 macrophages	Expression of Type I cytokines, anti-tumor effect
	M2 macrophages	Expression of Type II cytokines, inhibition of immune cells
Natural killer (NK) cells		Recognition and killing of tumor cells, cytokine secretion
B cells		Antibody secretion, processing and presentation of antigens, cytokine secretion
T cells	CD8^+^ T cells	Recognition and killing of tumor cells, cytokine secretion
	CD4^+^ T cells	Assisting CD8^+^ T cells, cytokine secretion
Myeloid-derived suppressor cells (MDSCs)		Inhibiting T-cell function and promoting the development and maturation of regulatory T cells (Tregs)

This table summarizes “Components of the TIME,” “Immune effects.” “Components of the TIME” means important cells or structures in the TIME. “Immune effects” means how do these components modulate immunity in the TIME.

### 2.1 DCs

DCs, originated from multipotent hematopoietic stem cells in the bone marrow, showing the remarkable ability to efficiently capture, process, and present antigens to T cells (Balan et al., 2019). After the activation of DCs, DCs showed increased costimulatory molecule expression, cytokine secretion, and antigen capture presentation ability (Veglia and Gabrilovich, 2017). Mature DCs can express major histocompatibility complex (MHC) and costimulatory molecule to effectively activate T cells, thus initiating the tumor-killing process of T cells (Banchereau et al., 2000; Gardner et al., 2020). Moreover, immunosuppressive factors on DCs are the key regulator of T cell immunity in the TIME (Oh et al., 2020). However, it was found that there were defects in the differentiation and activation of DCs in the TIME, which led to the inability of DCs to perform the immune surveillance function well (Veglia and Gabrilovich, 2017). Elevated lactate levels, hypoxia, adenosine accumulation, and decreased pH in the TIME affect the maturation and activation of DCs, which finally affect the antigen presentation ability of DCs (Veglia and Gabrilovich, 2017).

### 2.2 Macrophages

Macrophages can mediate phagocytosis and present antigens. Macrophages can differentiate into M1 and M2 macrophages, which exert an inverse influence on the progression of the tumor. M1 macrophages express Type I cytokines with an anti-tumor effect, such as tumor necrosis factor-α (TNF-α), interferon-γ (IFN-γ), IL-6; M2 macrophages express Type II cytokines, which inhibit T cell-mediated anti-tumor response, such as IL-10, IL-13, transforming growth factor-β (TGF-β) (Biswas and Mantovani, 2010). These cytokines can regulate TIME. For example, TNF-α can not only activate immune cells, but also promote tumor angiogenesis and immunosuppression in some cases (Cruceriu et al., 2020). M2 macrophages are the main type in the TIME, called TAMs, and help establish an immunosuppressive environment. And TAMs can promote the proliferation and metastasis of tumor cells by inhibiting the recognition and killing function of tumor cells by T cells and NK cells (Ngambenjawong et al., 2017; Qian and Pollard, 2010).

### 2.3 NK cells

NK cells, as the innate cytotoxic lymphocytes, are capable of recognizing and killing tumor cells without tumor antigens’ stimulation (Bald et al., 2020). Many tumor cells evade the immune recognition and killing of CD8^+^ T cells by down-regulating the expression of MHC-I. NK cells can recognize the “missing-self” phenotype of tumor cells (Myers and Miller, 2021; Kärre, 2002). Once NK cells are activated, NK cells can kill tumor cells by secreting perforin and granzyme. Moreover, NK cells can also exert anti-tumor immunity by mediating antibody-dependent cell-mediated cytotoxicity (ADCC) (Myers and Miller, 2021). For example, NKG2D serves as an activating receptor for NK cells, and its corresponding ligand is expressed in some tumor cells. When the NKG2D receptor binds to the ligand, it can trigger the killing function of NK cells in the TIME (Wang et al., 2020). Additionally, some cytokines and chemokines (IFN-γ, GM-CSF) can be secreted by NK cells, which have immunomodulatory and anti-tumor effects in the TIME.

### 2.4 B cells

B cells in the TIME are capable of regulating tumor cell survival and proliferation. Tertiary lymphoid structures (TLSs) can be formed in the TIME (Fridman et al., 2022). B cells in TLSs form germinal centers and secrete antibodies that can recognize tumor-associated antigens; B cells are also involved in antigen presentation in the TIME to activate immune response (N. et al., 2021; Chandnani et al., 2024). Further, B cells can also produce various immune-stimulating cytokines, including lymphotoxin and other cytokines (Tang et al., 2017).

### 2.5 T cells

Adaptive immunity mediated by CD8^+^ and CD4^+^ T cells is the leading force for killing tumor cells (Fridman et al., 2012). CD8^+^ T cells recognize MHC-I, while CD4^+^ T cells have an affinity for MHC-II (Raskov et al., 2021). CD8^+^ T cells can secrete particles containing granzymes, perforin, and cathepsin C to induce apoptosis of tumor cells (Farhood et al., 2019). In addition, activated CD8^+^ T cells can highly express Fas ligand (FasL), which can mediate Fas activation to induce target cell apoptosis binding to Fas (Fu et al., 2016). However, tumor cells can inhibit the killing effect of CD8^+^ T cells by reducing the level of MHC-I molecules on the cell membrane (Raskov et al., 2021). Another important type of T cells is CD4^+^ T cells. CD4^+^ T cells can secrete cytokines (IFN-γ, IL-4, IL-10, etc.) to regulate the immune response. Moreover, the subpopulations of CD4^+^ T cells have many functions and mechanisms in the TIME, which are playing the anti-tumor or pro-tumor role. T helper 1 (Th1) cells can promote recruitment of antigen-presenting cells, activate phagocytosis of macrophages, and inhibit angiogenesis; Th2 cells can inhibit antigen processing of DCs, promote polarization of M2 macrophages, activate immunosuppressive Tregs and Myeloid-derived suppressor cells (MDSCs), and activate the anti-tumor activity of NK cells; Th17 cells can promote angiogenesis (Miggelbrink et al., 2021; Ellyard et al., 2007; Basu et al., 2021). Tregs have the ability to inhibit anti-tumor immune response, thereby accelerating the process of tumor progression (Li C. et al., 2020). It is worth noting that there are other cells and molecules in the TIME that can inhibit effective anti-tumor immune responses, such as MDSCs, programmed cell death protein 1 (PD1), adenosine, and vascular endothelial growth factor (VEGF) (Cekic et al., 2014; Mannino et al., 2015; Keir et al., 2008; Pan et al., 2010).

### 2.6 MDSCs

Myeloid-Derived Suppressor Cells (MDSCs) are a type of immature myeloid cells with immunosuppressive functions, playing a crucial role in the tumor immune microenvironment (Veglia et al., 2021). They exert potent immunosuppressive effects by inhibiting T-cell function and promoting the development and maturation of regulatory T cells (Tregs). In the tumor immune microenvironment, MDSCs play a pivotal role in fostering tumor growth, progression, and metastasis by suppressing antitumor immune responses (Yaseen et al., 2020).

The immunosuppressive mechanisms are multifaceted, involving the production of immune-inhibitory cytokines such as interleukin-10 (IL-10), transforming growth factor-β (TGF-β), and arginase-1, which deplete essential amino acids required for T cell proliferation and function. Additionally, MDSCs express programmed death-ligand 1 (PD-L1), which binds to PD-1 on T cells, further inhibiting their activity. Moreover, MDSCs promote tumor angiogenesis by secreting vascular endothelial growth factor (VEGF), thereby providing nutritional support to the tumor. They also enhance tumor invasiveness by secreting matrix metalloproteinases (MMPs) and induce the polarization of macrophages towards the M2 phenotype, which further amplifies the immunosuppressive microenvironment (Gabrilovich and Nagaraj, 2009).

MDSCs play a crucial role in the tumor immune microenvironment by suppressing antitumor immune responses and promoting tumor progression. Understanding the complexities of MDSC biology and developing effective therapeutic strategies to target these cells hold great promise for improving cancer treatment outcomes (Ozbay Kurt et al., 2023).

## 3 Emerging immunomodulators of small molecule

After gaining a preliminary understanding of the composition of TMIE, which drugs might have a regulatory effect on it, thereby achieving activation of antitumor immunity. The past few years have witnessed an enormous interest in immunomodulatory agents to restrain tumors, especially drugs for treating other diseases, small molecules targeting drugs through chemical synthesis, and agonists of immune signaling screened from the compound library. Table 2 lists “Effective concentration” “Research model” “Administration” “Effect on immune cells” and “Mechanism” of emerging small molecular immunomodulators in tumors.

**TABLE 2:** Summary of the *in vitro* and *in vivo* evidence for small molecule immunomodulators.

Compound	Effective concentration	Research model	Administration	Effect on immune cells	Mechanism	Ref
Tramadol	20 and 40 mg/kg	Lung metastatic colonization model of rat	s.c.	Counteract the inhibition of NK cell activity and lymphocyte proliferation induced by surgery	n/a	Wang et al. (2024)
Tramadol	100 mg per patient	Patients undergoing modified radical mastectomy	i.v.	Increase the expressions of peripheral blood T cell and NK cell in tramadol group than morphine group	n/a	Bakr et al. (2016)
Tramadol	2.5, 5, and 10 mg/kg	Liver orthotopic tumor of C57BL/6 mice	By osmotic pump	Facilitate M1 macrophage polarization, and promote proliferation and activation of T cell	Facilitate M1 macrophage polarization by activating nuclear factor kappaB (NF-κB) signaling	Wang et al. (2024)
	2.5, 5.0 and 10.0 mg/L	BMDMs and THP-1 macrophages	*in vitro*			
Clotrimazole	20 μM	Coculture of B3Z or OT-I cells and DC2.4 or BMDCs pretreated with clotrimazole	*in vitro*	Enhance DC-mediated antigen presentation and promote T cell activation	Enhance DC-mediated antigen presentation by modulating lactate metabolic production, lysosome pathway, and CHOP expression in DCs	Wang et al. (2021)
	40 mg/kg	Bearing MC38 tumors model of C57BL/6 mice, nude mice, and *Batf3* ^*−/−*^ mice	i.p.			
		Bearing CT26 tumors model of Balb/c mice				
		Bearing B16F10 tumors model of C57BL/6 mice				
Clotrimazole	200 mg/kg	Bearing B16F10 tumors model of C57BL/6 mice	i.g.	Attenuate infiltration of immunosuppressive tumor-associated macrophages (TAM)	n/a	Ochioni et al. (2021)
Guanabenz, clonidine and guanfacine	5 and 10 μM	Coculture of CD8^+^ T cells and L1210.P1A.B7-1 cells	*in vitro*	Active macrophages, recruit and activate T cells in the TIME, and reduce myeloid-derived suppressor cells (MDSCs)	n/a	Zhu et al. (2023)
	5 mg/kg (guanabenz, clonidine) 2 mg/kg (guanfacine)	Bearing SK-OV-3, LS411N or PC-3 tumor model of NSG mice followed by being reconstituted with human lymphocytes	i.p.			
		Bearing MC38-OVA tumors of C57BL/6 mice received adoptive cell transfer of TCRP1A CD8^+^ T cell				
		Bearing MC38 tumors of C57BL/6 mice received adoptive cell transfer with *in vitro* differentiated macrophages				
		*Adra2a*-KO mice				
MSA-2	50 mg/kg	CT26, H22, and B16F10 subcutaneous tumor model and EMT-6 orthotopic tumor model	i.g.	Promote DC maturation, enhance BDMC activity, trigger proliferation of naïve T cells, and increase M1-like markers of BMDM rather than M2-like marker	Promote DC maturation by stimulating pro-inflammatory cytokines secretion and activating the type Ⅰ Interferon (IFN) signaling pathways	Yi et al. (2022)
	0.01 mg/mL	BMDCs for one-way mixed lymphocyte reaction (MLR) and OVA peptide pulse assay	*in vitro*			
ABBV-CLS-484	1, 3, 10, and 100 mg/kg	Bearing MC38 tumors model of C57BL/6 mice,	i.g.	Improve the activation and functions of NK cells, increase CD8^+^ T cells, prevent T cell exhaustion, and improve effector function and fitness of T cell	n/a	Baumgartner et al. (2023)
	15 and 300 mg/kg	Rat for toxicity study	i.g.			
	20 and 40 mg/kg	Mice cured MC38 tumors following AC484 treatment rechallenge MC38 subcutaneous tumors	i.g.			
	10, 30, and 100 mg/kg	C57BL/6 mice for dose-escalating mouse pharmacokinetics	i.g.			
	Twice daily at 10 mg/kg or once daily at 20 mg/kg or 100 mg/kg	Bearing B16F10 tumors model of mice vaccinated with GM-CSF-secreting B16 (GVAX) cells	i.g.			
	50, 100, and 150 mg/kg	Bearing B16F10 tumors model of C57BL/6 mice	i.g.			
	100 mg/kg	B16F10 pulmonary metastasis model	i.g.			
	20 and 60 mg/kg	4T1 metastasis model	i.g.			
	100 nM	Bearing EL4-OVA tumors of C57BL/6 mice received adoptive cell transfer of ABBV-CLS-484 treated OT-I CD8^+^ T cell	n/a			
	0.1 and 1 µM	*Ptpn2* or *Ptpn1* single deletion, or *Ptpn2/n1* dual deletion B16 cells	*in vitro*			
	1 and 20 μM	CD8^+^ T cells from mice for *in vitro* repeat antigen stimulation	*in vitro*			
	0.001, 0.1, 1 and 10 µM	Coculture of OT-1 CD8^+^ T cells and B16-OVA melanoma cells pretreated with ABBV-CLS-484	*in vitro*			
	0.1 and 1 µM	Coculture of A375 melanoma cells and T cells from human pretreated with ABBV-CLS-484	*in vitro*			
	0.1, 1 and 10 µM	BMDC and BMDM	*in vitro*			
	20 μM	CD8^+^ T for TCR signaling analyses	*in vitro*			
	0.1 and 1 µM	Coculture of NK cells and YAC-1 tumor cells	*in vitro*			
	0.001, 0.1, 1 and 10 µM	Pan T cells	*in vitro*			

This table summarizes “Effective concentration,” “Research model,” “Administration,” “Effect on immune cells,” and “Mechanism” of the above immunomodulators. “Effective concentration” means the specific dose level chosen to investigate the effects of immunomodulators on immune cells and their mechanisms. “Research model” means the specific type of experimental model used in the study. “Administration” means the deliver process of these immunomodulators and covers two different delivery methods *in vitro* and *in vivo*. “Mechanisms” refers to the molecular mechanisms these immunomodulators employ in immune cells.

### 3.1 Tramadol

Tramadol (TRA) is a weak opioid anesthetic and is prescribed for pain in tumors where non-opioid agents do not provide effective pain relief. Intriguingly, TRA has presented the anti-tumor ability in multiple tumors, such as pancreatic ductal adenocarcinoma (Kuramochi et al., 2024), endometrial carcinoma (Liu L. C. et al., 2022), lung adenocarcinoma (Xia et al., 2016a), breast cancer (Xia et al., 2016b). Moreover, compared to other opioids, tramadol could preserve the immune function, counteracting the inhibition of NK cell activity and lymphocyte proliferation induced by surgery for rats (Gaspani et al., 2002) and patients (Bakr et al., 2016) in the lab and clinical, respectively. In addition, TRA could facilitate M1 macrophage polarization by activating nuclear factor kappa B (NF-κB)signaling, thereby promoting the proliferation and activation of T cells (Wang et al., 2024). In light of evidence that tramadol showed protective immune function against tumors, prescription of opioids, particularly tramadol, could be recommended.

### 3.2 Clotrimazole

Clotrimazole (CTZ), an antifungal drug, is also an anti-tumor agent owing to its multifactorial function, including a pro-immune effect. CTZ triggered DC-mediated antigen presentation by modulating lactate metabolic production, lysosome pathway, and CHOP expression in DCs, subsequently promoting T cell activation (Wang et al., 2021). Moreover, CTZ could reprogram TIME by attenuating the infiltration of immunosuppressive TAMs, leading to an anti-tumor effect (Ochioni et al., 2021).

### 3.3 Statins

Statins, specifically inhibiting HMG-CoA reductase, are widely prescribed to reduce cholesterol. The literature has demonstrated that statins, including mevastatin, simvastatin, and pitavastatin, could enhance the tumor-killing effect mediated by PBMC (Selvin et al., 2023). Furthermore, T cells pretreated with simvastatin and lovastatin presented an increased cytotoxic effect on tumor cells. Daily oral administration has enhanced tumors’ sensitivity to PD-1 therapy and promoted the shift to M1 macrophage (Kansal et al., 2023; Vesely et al., 2022).

### 3.4 Agonists of α2-adrenergic receptors

Agonists of α2-adrenergic receptors (α2-AR), including guanabenz (GBZ), clonidine (CLD), and guanfacine (GFC), have been identified with pro-immune function and anti-tumor effect. These agonists could activate macrophages, leading to the recruitment and activation of T cells in the TIME. The number of PMN-MDSC was reduced in both the MC38-OVA model and the TiRP model, while macrophages played a prominent role in the MC38-OVA model. Moreover, these agents enhance the anti-tumor effect of anti-PD-1 therapy (Zhu et al., 2023).

### 3.5 MSA-2

Systemic non-nucleotide STING agonist, MSA-2, which was screened from a compound library for oral administration, regressed tumor growth and presented durable anti-tumor immunity against reinoculation of tumor (Pan et al., 2020). Additionally, synergized with anti-PD-1 and osimertinib, MSA-2 could enhance the anti-tumor effect and reverse the resistance of this therapy (Pan et al., 2020; Lin et al., 2023; Li et al., 2024; Yi et al., 2022). Furthermore, MSA-2 stimulated pro-inflammatory cytokines secretion and activated the type I Interferon (IFN) signaling pathways in human monocytes (Kabelitz et al., 2022). IFN could promote DC maturation. MSA-2 triggered DC maturation and significantly enhanced mice BDMC activity, including increased cytokine secretion and antigen presentation capability. BMDCs treated with MSA-2 triggered the proliferation of naïve T cells. Besides, MSA-2 also could increase M1-like markers of BMDM rather than M2-like marker (Yi et al., 2022).

### 3.6 BPR1R024

Based on physicochemical property-driven optimizations from a multitargeting kinase inhibitor, BPR1R024 was designed for oral BPR1R024 administration and presented superior target selectivity to CSF1R. BPR1R024 dramatically inhibited M2 macrophage growth while maintaining minimal effect on M1 macrophage, leading to an anti-tumor effect *in vivo* (Lee et al., 2021).

Besides, a broad range of studies has demonstrated that compounds derived from natural products, such as Fucoidans (Kiselevskiy et al., 2022), Humulus lupulus (Vesaghhamedani et al., 2022), mushrooms (Balakrishnan et al., 2021), Huang qi (Balakrishnan et al., 2021), and honey (Masad et al., 2021), present potent anti-tumor immunity, indicating that dietary therapy is like to improve individual self-defense against tumors.

Generally, diverse immunomodulators have huge potential to inhibit tumor progression with canonical chemotherapy (Deng et al., 2022) and radiotherapy (Ni et al., 2022), function as adjuvant to tumor vaccines (Chen F. et al., 2023), reverse the resistance to anti-tumor therapy (Baumgartner et al., 2023), and preserve immune function after surgery.

## 4 Hydrogel in the regulation of TMIE

**FIGURE 1:**
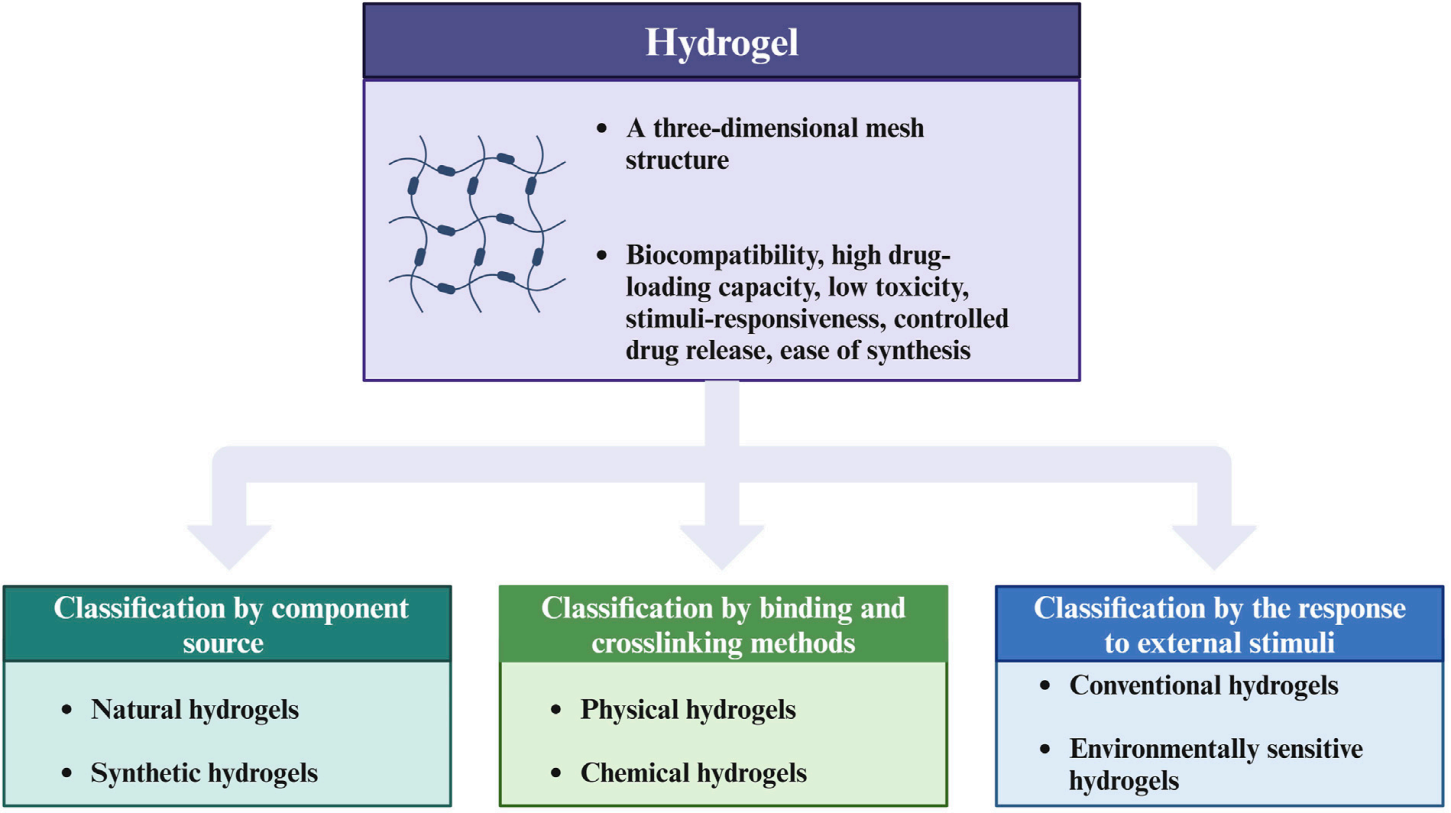
Classification chart of hydrogels. This figure introduces the advantages of hydrogels as biomaterials and summarizes three main classification methods of hydrogels, including classification by component source, by binding and crosslinking methods, and by the response to external stimuli. The figure is made with Biorender (https://biorender.com/).

As an emerging biomaterial, What advantages do hydrogels possess that enable them to play a role in biomedicine, particularly in the regulation of TMIE? Hydrogels have different kinds and can be classified according to different classification methods. Hydrogels can be categorized into natural and synthetic hydrogels according to the component source. The former include fibrous proteins, alginates, etc. The latter include polyvinyl alcohol (PVA) and polyethylene glycol (PEG) (Cui et al., 2021). Moreover, physical and chemical hydrogels are classified according to binding and crosslinking methods (Ahmed, 2015). Physical hydrogels are formed by the combination of physical forces. Chemical hydrogels are formed by the crosslinking of chemical bonds. Classification based on response to external stimuli includes conventional and environmentally sensitive hydrogels. Traditional hydrogels are not sensitive to external stimuli, while environmentally sensitive hydrogels can respond to external stimuli (such as pH, temperature, pressure, and light) (Li and Su, 2018). In addition, hydrogels can also be classified according to the polymer composition (Ahmed, 2015). Figure 1 presents the classification of hydrogels.

Nowadays, hydrogels are widely used in biomedicine (including tissue engineering, drug delivery, 3D cell culture, wound dressing, etc.) (Ho et al., 2022), agriculture (Khan et al., 2022), and industrial materials (Flores-Valenzuela et al., 2023). Especially in medicine, hydrogels have presented specific feasibility and effectiveness for the treatment of anal fistulas (de la Portilla et al., 2021), gingival regeneration (Hutomo et al., 2023), wound dressing (Liang et al., 2021), and burn wound management (Goh et al., 2024), etc. Hydrogels can provide the loaded immune components with a niche similar to the environment *in vivo* due to their excellent biocompatibility, 3D network structure. Good biocompatibility means less toxicity for cells. The 3D network structure can make hydrogels have high permeability, allowing the transport of nutrients and the transfer of signaling molecules, ultimately promoting cell metabolism and proliferation (Yue et al., 2020). And highly hydrophilic and porous structure allows hydrogel to absorb and store large amounts of water, mimicking the ECM. Besides, this moist environment supports the wound healing (Zhang and Zhao, 2020). Moreover, hydrogels can promote wound healing via carrying growth factors and antibiotics, which are continuously released on the wound. In bone tissue engineering (BTE), hydrogels provide scaffolds for growth factor transport and cell adhesion (Yue et al., 2020). Hydrogels are injected or surgically implanted to fill complex bone defects (Xue et al., 2022; Buwalda et al., 2017).

Hydrogel’s 3D network mechanism can efficiently deliver immunomodulators to the tumor site and maintain a high concentration for a long time, while significantly reducing the incidence of systemic toxicity and the risk of adverse side effects during immunotherapy (Chao et al., 2020). Based on hydrogels, anti-tumor immune responses can be induced by targeting immune checkpoint molecules and factors. Among them, the typical are PD-1/PD-L1 (Shi et al., 2021), CTLA-4 (Chen W. et al., 2023), NKG2D (Wang et al., 2020), TLRs (Behzadi et al., 2021), TGF-β (Gulley et al., 2022), etc. For example, antiprogrammed cell death receptor ligand 1 (aPD-L1) released by the hydrogel inhibited the PD-1/PD-L1 pathway and restored cytotoxic T cell killing function at the tumor site (Shi et al., 2021). Hydrogels can also efficiently deliver DCs, NK cells, and T cells to the tumor site. Moreover, the stimulation response of the intelligent hydrogel enables the hydrogel to release these components at the tumor site accurately, improving the targeting and anti-tumor effect (Zhao et al., 2022). In summary, hydrogels have demonstrated significant potential in the tumor treatment.

### 4.1 Targeted modulation of DCs by hydrogels

**FIGURE 2:**
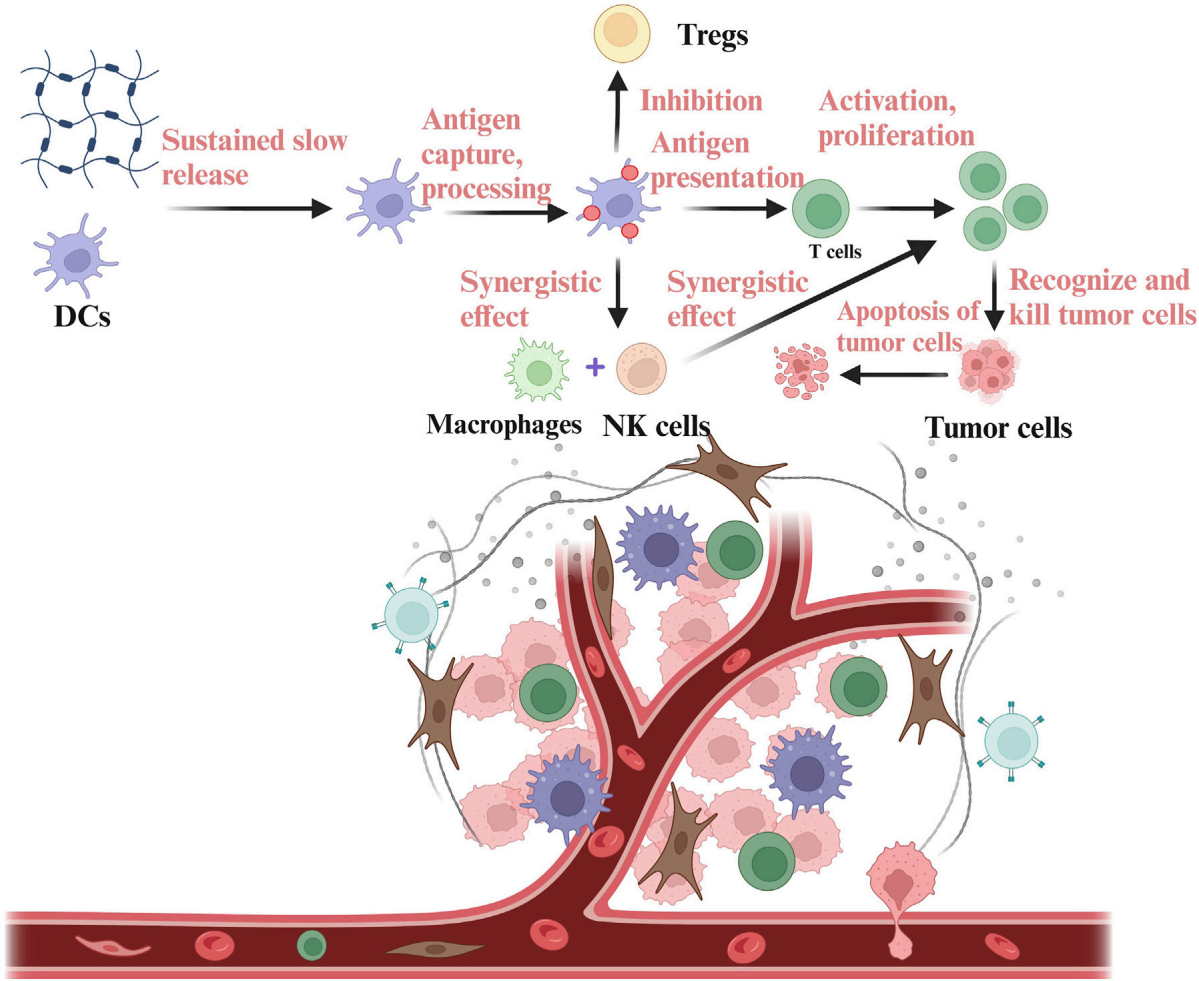
Delivery of DCs based on hydrogels in the TIME. DCs are encapsulated in the hydrogels and then released into the TIME. DCs released into the TIME can carry out antigen capture, processing and presentation, thus stimulating the anti-tumor immune function of T cells. DCs can also co-mediate immune response with NK cells and macrophages, while inhibiting immunosuppressive components in the TIME, such as Tregs. The figure is made with biorender (https://biorender.com/).

DC vaccines have shown a positive effect on the strategy of tumor immunotherapy and have achieved good results in clinical therapy (Palucka and Banchereau, 2013; Timmerman et al., 1999). However, DCs have a high mortality rate and a high off-target risk during the process of reaching the site of the tumor (Palucka et al., 2007). So, it is often challenging to recruit sufficient dendritic cells (DCs) for uptake, processing, and presentation. By leveraging the properties of hydrogels, antigens and immunostimulants can be encapsulated, along with the addition of pro-inflammatory cytokines and chemokines. This approach enables the sustained release of antigens, immunostimulants, cytokines and chemokines, thereby enhancing antigen presentation, promoting inflammatory responses, increasing DC recruitment, and inducing a robust anti-tumor immune response (Lei et al., 2022). DCs work synergically with macrophages and NK cells to exert immune function and inhibit the function of Tregs, ultimately activating T cells to recognize and kill tumor cells. For example, a biomaterial system of alpha-cyclodextrin-polyethylene glycol hydrogel/CpG crosslinked nano-adjuvants was used to co-deliver tumor cells and DC vaccines (Yang et al., 2021), and subsequently recruited DCs to the tumor site (Figure 2).

### 4.2 Targeted modulation of macrophages by hydrogels

TAMs are the main type of macrophages in the TIME. TAMs can support the growth and metastasis of tumor cells by inhibiting innate and adaptive immune cells and promoting angiogenesis. Tumor-associated macrophages (TAMs) can be targeted by depleting their numbers, repolarizing their function, or activating their ability to phagocytose tumor cells (Pathria et al., 2019). In tissue regeneration, a study found that the fibrin hydrogel may induce the anti-inflammatory phenotype of macrophages to eliminate the inflammatory response, leading to massive macrophage infiltration in the hydrogel scaffolds (Tanaka et al., 2019). This can also be applied to tumor therapy, but the direction of polarization needs to be transformed to M1 macrophages. Moreover, NF-κB signaling helps maintain the immunosuppressive phenotype of TAMs. Targeting NF-κB signaling in TAMs can inhibit tumor growth by enhancing macrophage tumoricidal activity and recruiting IL-12-dependent NK cells (Hagemann et al., 2008). Therefore, regulation of NF-κB based on hydrogels maybe a potential strategy.

### 4.3 Targeted modulation of NK cells by hydrogels

NK cells can inhibit tumor progression via anti-angiogenesis and proapoptotic effects (Myers and Miller, 2021). However, due to the complexity of the TIME, NK cell infiltration is less effective in immunotherapy for some solid tumors (Sun et al., 2015). Immunotherapy can increase NK cell infiltration to enhance immunosurveillance. It has been found that hydrogels loaded with immunomodulators can promote the infiltration of NK cells to induce the apoptosis of tumor cells more efficiently. A double pH-responsive hydrogel containing tumor acidity neutralizer and neutrophil extracellular traps (NETs) lyase in combination with NK cell infusion was used to prevent the recurrence of hepatocellular carcinoma (HCC) following resection (Cheng et al., 2022). Tumor acidity neutralizer effectively reduced the infiltration degree of immunosuppressive cells in the TIME while NETs lyase degraded NETs, which also enhanced NK cell infiltration. Hydrogels can also target NK cells by delivering and releasing anti-TIGIT monoclonal antibody (aTIGIT) to increase the amount of NK cells. A study exhibited that the MMP-2 degradable hydrogel promoted the formation of immunogenic TIME and reversed the exhaustion of NK cells and effector T cells by releasing DOX and aTIGIT (Tian et al., 2022), thus depressing tumor growth. In addition, targeting NKG2D based on hydrogels is an important way to activate the anti-tumor activity of NK cells in the TIME (Jiao et al., 2024).

### 4.4 Targeted modulation of MDSCs by hydrogels

MDSCs are a heterogeneous population, exerting immunosuppressive function in the TIME. MDSCs inhibit immunity in the TIME through reactive oxygen species (ROS), production of nitric oxide (NO), TGF-β, PD-L1 expression, and so on (Li et al., 2021). Multiple signal transduction pathways enhance the infiltration of MDSCs in the TIME, such as CXC chemokine ligand 13/CXC chemokine receptor type 5 (CXCL13/CXCR5) signaling, and chemokine ligand 15/chemokine receptor 1 (CCL15/CCR1) signaling (Li B.-H. et al., 2020). For example, CXCL13/CXCR5 can participate in MDSC recruitment and help tumor cells escape T cell immunity (Li B.-H. et al., 2020; Hussain et al., 2019). In addition, IL-6 is regarded as a major regulator of MDSC accumulation and activation, which is capable of promoting tumor cell proliferation and metastasis (Weber et al., 2021). Therefore, regulating the above signaling pathways or molecules maybe the important methods for targeting MDSCs.

### 4.5 Targeted modulation of T cells by hydrogels

The downregulation of MHC-I of tumor cells impedes the recognition for CD8^+^ T cells, leading to immune escape. Hydrogels loaded with immunomodulators can promote the recognition of tumor cells by CD8^+^ T cells by upregulating MHC-I (Zhao et al., 2023). A study found that in the hydrogels, 4-1BB antibody promoted T cell mitochondrial biogenesis and the axitinib-lowered hypoxia synergistically reversed T cell exhaustion (Zhang et al., 2022). At the same time, PF-06446846 amplified MHC-I expression to improve the recognition of tumor cells by T cells.

**FIGURE 3:**
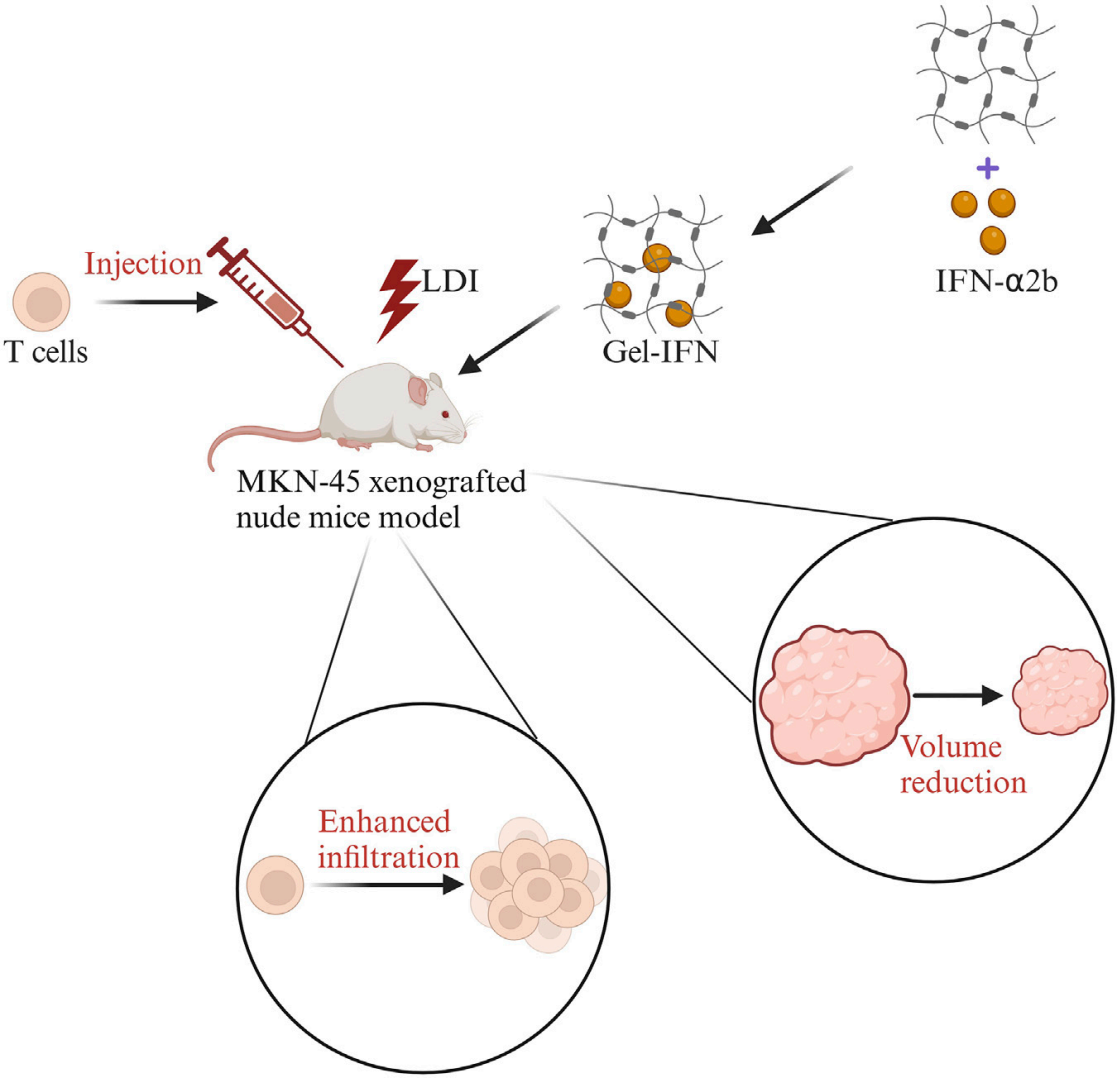
The sketch map of IFN-α2b hydrogels combined with T cell transfer and low-dose irradiation (LDI) in the treatment of gastric cancer. First, LDI was used in the MKN-45 xenografted nude mice model, then T cells were injected in the mice model, followed by IFN-α2b-loaded hydrogels (Gel-IFN). Finally, the enhanced infiltration of T cells and reduced tumor volume were observed. Modified from ref. Liu et al. (2020). The figure is made with biorender (https://biorender.com/).

The immune activity of T cells can be activated by hydrogels loaded with cytokines (Liu et al., 2020; Del Río et al., 2020). Cytokines are soluble low molecular weight proteins secreted by T cells, B cells, NK cells, etc. IFN-α can regulate the proliferation and differentiation of T cells and increase the level of IFN-γ to promote apoptosis of tumor cells (Brinkmann et al., 1993). IFN-α can also promote the expression of MHC-I molecules and enhance antigen presentation to improve the recognition efficiency of tumor cells. IFN-α2b is an important subtype of IFN-α. A hydrogel encapsulated with the enhancer IFN-α2b in combination with T cell transfer and low-dose irradiation (LDI) maintained a high activity of IFN-α2b and enhanced the recruitment of T cells in the TIME (Liu et al., 2020). Finally, the tumor volume was reduced. The specific process is shown in Figure 3. Besides, the hydrogel loaded with CCL21 was found that it can recruit T cells for homing and infiltration (Del Río et al., 2020). Therefore, hydrogels can solve the challenges of low immune efficiency caused by insufficient expansion of T cells in tumor immunotherapy (Fesnak et al., 2016).

Targeting immune checkpoints based on hydrogels is also a very important mechanism. Hydrogels can deliver immune checkpoint inhibitors to enhance the immune activation of T cells. Here, we mainly focus on the PD-1/PD-L1 checkpoint pathway. PD-1 is an important immunosuppressive molecule that can be expressed on the surface of activated T cells and B cells (Keir et al., 2008). When PD-1 binds to its ligand PD-L1, it produces inhibitory signals that inhibit the proliferation of T cells, cytokine release, and killing effect (Liu et al., 2021; Han et al., 2020). This binding can also initiate the programmed death of T cells (Liu et al., 2021; Han et al., 2020). Therefore, blocking the PD-1/PD-L1 pathway can restore immune surveillance and promote the recognition and killing of tumor cells by T cells. Moreover, T cell therapy combined with PD-1/PD-L1 checkpoint inhibitors can produce an obvious tumor cell-killing effect. For example, a hydrogel loaded with CAR-T cells, human platelets coupled with aPD-L1, IL-15, *etc.*, can reshape the TIME and promote CAR-T cell infiltration (Hu et al., 2021).

Furthermore, L-arginine plays a crucial role in the proliferation, differentiation, and survival of T cells, enhancing their anti-tumor immune response (Geiger et al., 2016). Arginase1 (ARG1) exhibits immunosuppression by reducing the level of L-arginine (Geiger et al., 2016). Norvaline is an inhibitor of ARG1, and hydrogels can provide a powerful platform for norvaline to block the ARG1 pathway to regulate T cells. Hydrogels loaded with norvaline and doxorubicin hydrochloride achieved high drug loading and controlled release of norvaline to block the ARG1 pathway, showing promising efficacy in primary tumor ablation and inhibition of tumor metastasis (Ren et al., 2021).

As an important subtype of T cells, Tregs can mediate inhibition through regulation of DCs, cytolysis of target cells, and secretion of anti-inflammatory cytokines (Tanaka and Sakaguchi, 2017; Kim et al., 2020). Moreover, Tregs can not only interact directly with T cells and NK cells, but also indirectly inhibit the anti-tumor activity of T cells and NK cells through producing immunomodulatory cytokines such as IL-10 and TGF-β (Liu Y. et al., 2022). In the TIME, Tregs can express cytotoxic T-lymphocyte-associated antigen 4 (CTLA-4) molecules to promote immune escape (Watanabe et al., 2023). Immunosuppression function of Tregs can be reversed by the hydrogel loaded with CTLA-4 antibodies. In summary, targeting Tregs based on hydrogels maybe also an important method of enhancing anti-tumor immune responses (Pitt et al., 2016).

### 4.6 Targeted modulation of B cells by hydrogels

At present, there are few studies on how hydrogels target B cells, but some studies have proved that hydrogels have a positive regulatory effect on humoral immunity. The study found that sustained exposure to vaccine components based on polymer nanoscale (PNP) hydrogels enhanced the degree and time of germinal center responses in lymph nodes, promoting antigen-specific antibody affinity (Roth et al., 2020). Finally, the magnitude, duration, and quality of humoral immunity were enhanced. It provides an important reference for regulating B cells in the TIME by hydrogels to enhance anti-tumor immune response.

### 4.7 Hydrogel platforms or formulations in cancer immunotherapy

Among the various delivery systems and platforms, hydrogel platforms and hydrogel-based formulations have demonstrated considerable potential in enhancing the efficacy and safety of cancer immunotherapy (He et al., 2024). Recent advancements in hydrogel technology have led to the development of more sophisticated formulations, such as stimuli-responsive hydrogels that can release therapeutic agents in response to specific cues, such as pH changes or enzymatic activity (Solanki and Bhatia, 2024; Andrade et al., 2021). As research in this field continues to evolve, we anticipate the development of even more innovative and personalized hydrogel-based therapeutic strategies that will further improve immunotherapy outcomes.

#### 4.7.1 TransCon hydrogel

TransCon hydrogel represents an innovative drug delivery system that merges the sustained-release properties and biocompatibility of hydrogels to achieve continuous and stable delivery of immunomodulators or other therapeutic agents directly to the tumor site (Lessmann et al., 2023). This system enhances drug bioavailability and minimizes systemic side effects by precisely regulating the rate and duration of drug release. In the context of cancer therapy, TransCon hydrogel is employed to administer a variety of immunomodulators, such as LR7/8 Agonist (Zúñiga et al., 2022) and IL-2 β/γ (Rosen et al., 2022), aiming to bolster the body’s immune response and facilitate the destruction of tumor cells. This targeted approach ensures that the therapeutic agents are delivered where they are most needed, optimizing their efficacy while reducing off-target effects.

#### 4.7.2 STM-416

STM-416 is an injectable, biodegradable hydrogel formulation containing the TLR7/8 agonist resiquimod has been developed for intra-tumoral extended-release. The hydrogel serves as a carrier, offering a stable environment for drug release and providing a layer of protection to the immunomodulator against degradation by the body’s internal environment, thereby enhancing both its therapeutic effect and safety profile. It undergoing Phase 1/2a Study for bladder cancer, when administered during bladder tumor surgery, it locally and sustainably releases resiquimod, which binds to TLR7 and eight on immune cells like dendritic cells, macrophages, and B lymphocytes. This activates the TLR signaling pathway, leading to the induction of NF-kB and other transcription factors, enhanced Th1 immune responses, and increased cytokine production, particularly INF-a. Activation of dendritic cells further stimulates CTL and B-lymphocyte responses, potentially causing tumor cell lysis.

Collectively, the application of hydrogel platforms and hydrogel/immunomodulator formulations in cancer treatment introduces new avenues for precision medicine. By precisely controlling the release and distribution of drugs, these technologies can more effectively activate the immune system, enabling precise targeting of tumor cells. This results in improved treatment outcomes, reduced side effects, and ultimately, a better treatment experience and quality of life for patients. As research in this field continues to advance, the potential for these innovative approaches to revolutionize cancer treatment becomes increasingly evident.

## 5 Conclusion

TIME (Tumor Microenvironment) exhibits an immunosuppressive state that favors tumor progression. Hydrogels stand out as exceptional biomaterials in the realm of tumor immunotherapy due to their unique combination of biocompatibility and degradability. Furthermore, hydrogels have the capability to locally deliver immunomodulators, minimizing side effects, releasing them in a controlled manner directly to the tumor site, and preserving their bioactivity. The utilization of hydrogels can enhance the infiltration of immune cells into the TIME and activate the immune responses of dendritic cells (DCs), natural killer (NK) cells, T cells, and other immune cells. The regulatory mechanisms of hydrogels on innate and adaptive immunity within the TIME are intricate and diverse. Gaining a deeper understanding of how hydrogels regulate these immune cells in the TIME provides valuable insights for the optimal design of hydrogels and paves the way for the development of more efficient and safer tumor treatment strategies in the future.

Looking ahead, it is anticipated that the regulatory mechanisms of hydrogels in the TIME will be further explored with the advent of new drugs and technologies.

### 5.1 Precise drug delivery and controlled release

In the future, the primary application of hydrogels in TIME immune regulation will be as a precise drug delivery system. By adjusting the chemical composition, crosslinking strategies, and physical structure of hydrogels, researchers can design responsive hydrogels that release drugs in response to specific biological and pathological stimuli (such as pH, temperature, reactive oxygen species, etc.). This precise controlled release capability will significantly enhance the efficiency and safety of immunotherapy, reduce systemic adverse reactions, and broaden the therapeutic window.

### 5.2 Enhancement of immune cell infiltration and activation

The application of hydrogels is expected to significantly improve the infiltration and activation of immune cells in the TIME. By mimicking the properties of the extracellular matrix (ECM), hydrogels can provide a microenvironment conducive to the growth and activation of immune cells. Furthermore, hydrogels can load and slowly release cytokines and chemokines, attracting and activating immune cells such as dendritic cells (DCs), natural killer (NK) cells, T cells, etc., thereby enhancing the anti-tumor immune response.

### 5.3 Induction of immune tolerance and reduction of immune rejection

In cell therapy, the use of allogeneic cells faces the challenge of immune rejection. Hydrogels offer an innovative solution to mitigate this rejection. By encapsulating transplanted cells within a biocompatible hydrogel matrix, an immune-isolating physical barrier can be formed between the allogeneic cells and the host, effectively protecting the transplanted cells from cell-cell contact-mediated immune reactions. This strategy is expected to improve the success rate of cell therapy and promote its clinical application.

### 5.4 Synergistic combination immunotherapy

Future research directions also include exploring the application of hydrogels in synergistic combination immunotherapy. Through the hydrogel platform, multiple immunomodulators or cell therapies can be delivered simultaneously to achieve synergistic therapeutic effects. This combination strategy is expected to overcome the limitations of monotherapy and improve the overall efficacy of tumor immunotherapy.

### 5.5 Integration of new materials and technologies

With the continuous development of materials science and biotechnology, more new materials and technologies will be applied to the design and preparation of hydrogels in the future. For example, the emergence of novel hydrogel materials such as supramolecular hydrogels and smart hydrogels will provide more functions and broader application prospects for TIME immune regulation. At the same time, the integration of advanced biomanufacturing technologies such as gene editing and nanotechnology will further promote the development of hydrogels in the field of tumor immunotherapy.

### 5.6 Personalized medicine and precision medicine

The future prospects for hydrogels in TIME immune regulation also include their application in personalized medicine and precision medicine. By combining factors such as the patient’s genetic information, tumor type, stage, and immune status, customized hydrogel treatment plans can be designed for individual patients. This personalized medicine strategy will further improve the specificity and effectiveness of tumor immunotherapy.

In summary, the future prospects for hydrogels in TIME immune regulation are filled with hope and challenges. With the continuous progress of science and technology and the deepening of clinical applications, it is believed that hydrogels will play an increasingly important role in the field of tumor immunotherapy, bringing more blessings to cancer patients.
